# Increased Interleukin-17F is Associated with Elevated Autoantibody Levels and More Clinically Relevant Than Interleukin-17A in Primary Sjögren's Syndrome

**DOI:** 10.1155/2017/4768408

**Published:** 2017-01-22

**Authors:** Yuzhou Gan, Xiaozhen Zhao, Jing He, Xu Liu, Yun Li, Xiaolin Sun, Zhanguo Li

**Affiliations:** ^1^Department of Rheumatology and Immunology, Peking University People's Hospital and Beijing Key Laboratory for Rheumatism Mechanism and Immune Diagnosis (BZ0135), Beijing, China; ^2^Center of Clinical Immunology, Peking University, Beijing 100044, China; ^3^Peking-Tsinghua Center for Life Sciences, Beijing, China

## Abstract

Th17 related immune response is pathogenic in primary Sjögren's syndrome (pSS). However, the role of IL-17F, one potent inflammatory member of IL-17 family cytokines in pSS, has not been specifically defined. We recruited one hundred and nine pSS patients and forty-two healthy controls and their serum levels of IL-17A and IL-17F were determined by multiplex cytokine assays. White blood cell, red blood cell, neutrophil, lymphocyte, IgM, IgG, C3, C4, RF, ANA, anti-SSA antibody, and anti-SSB antibody were measured by standard laboratory techniques. EULAR Sjögren's syndrome disease activity index (ESSDAI) score was also evaluated accordingly. We found that IL-17F was significantly increased in pSS patients. Elevated levels of IL-17F were associated with increased IgG and IgM, higher titers of ANA and anti-SSA antibodies, and reduction of C3 and C4. Patients with higher disease activity also showed higher serum IL-17F levels. However, serum IL-17A was only increased in patients with longer disease duration and showed few correlation with clinical and laboratory features in pSS patients. In conclusion, IL-17F was correlated with increased autoantibody levels and disease activity in pSS and is more clinically relevant than IL-17A.

## 1. Introduction

Primary Sjögren's syndrome (pSS) is an autoimmune disease with exocrine gland dysfunction and at least one-third of pSS patients experience multiorgan involvement [1]. Historically, pSS was thought to be a Th1 driven disease due to the predominance of CD4^+^ T cells and their product, interferon-*γ*, in target organs and peripheral blood from these patients [2]. However, accumulating studies in human and animal models have revealed that Th17 immune response and IL-17 family cytokines also play a vital role in pSS [3–11].

IL-17A, commonly referred to as IL-17, is involved in normal physiological processes [12] and is also a leading pathogenic cytokine in a wide range of pathologic conditions, including cancer and autoimmune disorders, due to its strong proinflammatory effects [13]. Besides IL-17A, there are five other members structurally related to IL-17A in the IL-17 family, which are IL-17B, IL-17C, IL-17D, IL-17E (IL-25), and IL-17F. These molecules bear 20%–50% homology to IL-17A, especially within the C-terminal region [12].

IL-17F, first identified in 2001, shares the highest homology with IL-17A and is encoded by the* Il17f* gene in the same chromosome region of* Il17a* [14, 15]. IL-17F is mainly expressed by Th17 and IL-17-producing-*γδ*T cells [15]. Furthermore, IL-17F and IL-17A bind the same receptor complexes [16] and have similar functions in terms of induction of chemokines and cytokines [17] and activation of neutrophils and lymphocytes [18]. Although IL-17A and IL-17F are highly homologous, they do not always function in the same manner [19]. IL-17F is involved more in tissue inflammation and is shown to be a stronger neutrophil-recruiting cytokine than IL-17A [20]. In some diseases, such as psoriasis [21, 22] and asthma [23], IL-17F plays a more prominent role. However, there are no studies focusing on the clinical relevance and pathogenic roles of IL-17F in pSS and many other autoimmune diseases.

In the present study, we determined the serum levels of IL-17F in pSS patients comparing with healthy controls and analyzed their clinical relevance in pSS. It showed that increased IL-17F was positively associated with autoantibody levels and disease activity in pSS and might play a more dominant role in the pathogenesis of pSS in comparison to IL-17A.

## 2. Methods

### 2.1. Patients and Controls

We recruited 109 patients from the Department of Rheumatology, Peking University People's Hospital, Beijing, China, during January 2014 and December 2015. All were diagnosed with pSS and fulfilled the 2002 American-European Consensus Group Classification Criteria [24]. The exclusion criteria are (1) any other systemic autoimmune diseases; (2) severe infection, malignant tumor, severe organ dysfunction, or any other life-threatening conditions. We also recruited 42 healthy controls (HC) from the health examination center of the same hospital, with age and sex matched. All of them were excluded from any autoimmune diseases. The study was approved by the Ethics Committee of Peking University People's Hospital (Approval number 2015PHB219-01). All participants of this study had been informed and signed the consent for participation in this study.

### 2.2. Clinical and Laboratory Data

All patients underwent extensive clinical medical examinations and serological evaluations, including disease duration, lymphadenectasis, splenomegaly, white blood cell (WBC) count, neutrophil granulocyte count, lymphocyte count, red blood cell (RBC) count, hemoglobin (Hb), platelet count (PLT), immunoglobulin M (IgM), immunoglobulin G (IgG), complement 3 (C3), complement 4 (C4), rheumatoid factor (RF), erythrocyte sedimentation rate (ESR), antinuclear antibodies (ANA), and EULAR Sjögren syndrome disease activity index (ESSDAI) score [25]. Besides, joint involvement is defined as the existence of arthralgia or arthritis; pulmonary involvement is defined by the presence of respiratory symptoms (mainly persistent cough and/or dyspnoea) or altered pulmonary diagnostic tests (including pulmonary function tests (PFTs) and/or CT scan) [26].

### 2.3. Serum Samples

3 mL of peripheral venous blood was collected from both groups and waited to clot in room temperature for 2 hours. Samples were then centrifuged for 15 minutes at 3000 rpm under 4°C. The serum was then collected in polypropylene microfuge tubes and stored at −80°C for further analysis.

### 2.4. Measurement of Anti-SSA Antibody and Anti-SSB Antibody

Serum levels of anti-SSA antibody and anti-SSB antibody were measured by anti-SSA antibody IgG ELISA kit (Euroimmun) and anti-SSB antibody IgG ELISA kit (Euroimmun). The procedure was according to the manufacturer's instructions. Concentrations of anti-SSA antibody and anti-SSB antibody were determined by a linear standard curve.

### 2.5. Measurement of Th-17 Associated Cytokines

Serum levels of IL-17A and IL-17F were measured by MILLIPLEX MAP Human Th17 magnetic bead panel kits (Millipore, USA). The measurement procedure was based on the Luminex xMAP technology (EMD Millipore, USA), following the manufacturer's instructions. Interpanel and intra-assay control plasma samples were included to ensure consistency across panels. Data were read on the Luminex MAGPIX machine (Luminex Corporation) and analyzed using MILLIPLEX Analyst 5.1 software (Millipore). Concentrations of cytokines were calculated using a cubic standard curve.

### 2.6. Statistical Analysis

Data analyses were performed using SPSS 19.0 for Windows. The distribution of numerical data was evaluated by Shapiro-Wilk test. Numerical data with normal distribution were expressed as the mean ± standard and differences between two groups were analyzed by independent *t*-test. Numerical data with skewed distribution were expressed as median (P25, P75) and differences between two groups were analyzed by Mann–Whitney test. Ranked data were expressed as percentage and differences between two groups were analyzed by chi-square test. Correlations between variables were determined by Spearman's correlation coefficient. The cut-off values of IL-17A and IL-17F were determined by receiver operating characteristics (ROC) curve analysis. *P* < 0.05 was considered statistically significant.

## 3. Results

### 3.1. Characteristics of pSS Patients and Controls

A total of 109 pSS inpatients and 42 healthy controls with matched age and gender were included in this study. The mean age of 109 pSS patients at the time of our study was 55.72 ± 14.64 years (range 12–76) and the mean disease duration was 10.39 years ranging from 2 months to 40 years. Demographic, clinical, and laboratory features of pSS patients and healthy controls are shown in Table 1. Anti-SSA antibody and anti-SSB antibody were positive in 68 (62.85%) and 60 (55.06%), respectively. 62 pSS patients (56.88%) showed moderate to high disease activity.

### 3.2. Serum Levels of IL-17F Were Significantly Elevated in pSS Patients instead of IL-17A

**Figure 1:**
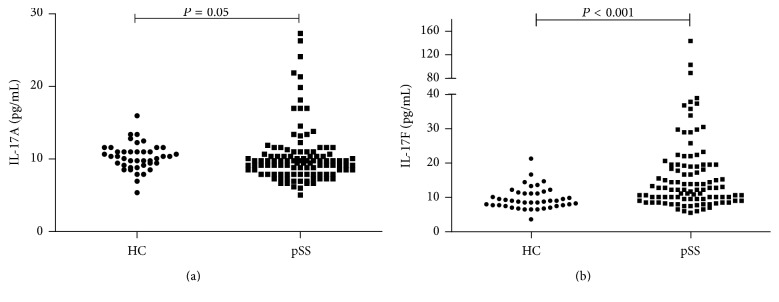
Comparison of serum levels of IL-17A and IL-17F between pSS patients and HC. (a) Serum IL-17A levels were not significantly elevated in pSS patients versus HC. (b) Serum IL-17F levels were significantly elevated in pSS patients versus HC.

As shown in Figure 1, the serum level of IL-17F (Figure 1(b)  20.35 ± 20.66 versus 9.56 ± 3.20, *P* = 0.001) was significantly higher in pSS patients comparing to healthy controls, while serum level of IL-17A (Figure 1(a)  10.37 ± 3.95 versus 10.29 ± 1.87, *P* = 0.05) did not show statistical significance in the two groups. According to the cut-off values for identification of overexpressed serum IL-17A and IL-17F, 109 pSS patients were grouped into IL-17F-elevated group (IL-17F ≥ 15.44 pg/mL, *n* = 43) and IL-17F-normal group (IL-17F < 15.44 pg/mL, *n* = 66) and IL-17A-elevated group (IL-17A ≥ 13.8 pg/mL, *n* = 12) and IL-17A-normal group (IL-17A < 13.8 pg/mL, *n* = 97).

### 3.3. Increased Serum Levels of IL-17F Were Associated with Elevated Autoantibody Level in pSS

As shown in Table 2, in pSS patients, IL-17F were positively correlated with RF (*r* = 0.481, *P* < 0.001) and anti-SSA antibodies (*r* = 0.236, *P* = 0.014). However, serum level of IL-17A was only positively correlated with RF (*r* = 0.234, *P* = 0.015). Besides, we divided pSS patients into the elevated group and the normal group, respectively, according to the cut-off values. As shown in Table 3, patients with higher serum levels of IL-17F showed elevated RF (205.00 (42.00, 1689.50) versus 20.00 (20.00, 69.50), *P* = 0.001), higher titers of anti-SSA antibodies (1484.99 (178.52, 1689.50) versus 454.25 (18.89, 1386.96), *P* = 0.002), and higher percentage of ANA ≥ 1 : 320 (70% versus 46.15%, *P* = 0.026), but patients with higher serum levels of IL-17A only showed higher titers of RF (345.50 (24.28, 573.25) versus 27.35 (20.00, 193.50), *P* = 0.016) and higher percentage of ANA ≥ 1 : 320 (72.73% versus 31.91%, *P* = 0.017).

Significantly elevated IL-17F levels were also observed in pSS patients with increased autoantibody levels compared with patients with lower autoantibody titers, which further support the positive correlation of IL-17F with pSS associated autoantibody levels. (Table 4).

### 3.4. IL-17F Was More Relevant with Increased pSS Severity and Disease Activity Than IL-17A

**Figure 2:**
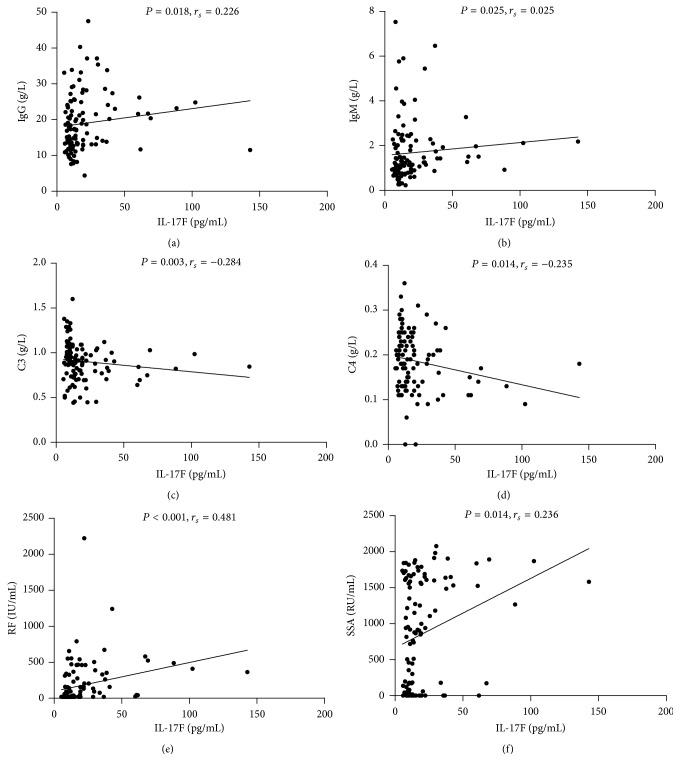
Correlation of serum level of IL-17F with laboratory parameters in pSS patients: (a) IL-17F positively correlated with IgG; (b) IL-17F positively correlated with IgM; (c) IL-17F negatively correlated with C3; (d) IL-17F negatively correlated with C4; (e) IL-17F positively correlated with RF. (f) IL-17F showed positive correlation with anti-SSA antibody. C3: complement 3.

**Figure 3:**
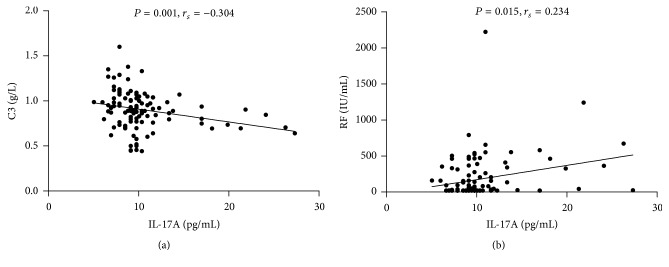
Correlation of serum level of IL-17A with laboratory parameters in pSS patients: (a) IL-17A were negatively associated with C3; (b) IL-17A were positively associated with RF.

**Figure 4:**
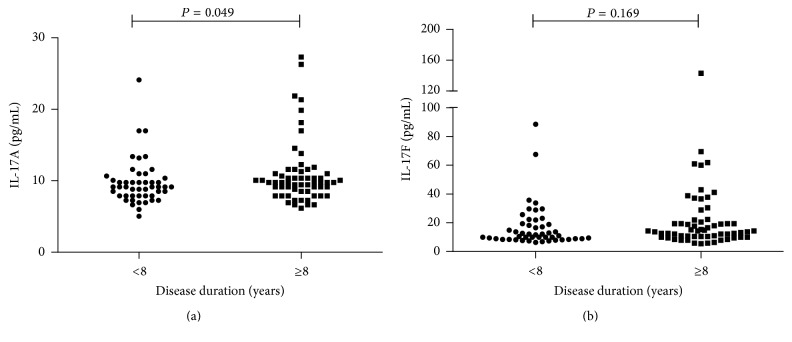
Comparison of serum levels of IL-17A and IL-17F between pSS patients with longer disease duration (≥8 years) and shorter disease duration (<8 years). (a) Serum IL-17A levels were significantly elevated in pSS patients with a longer disease duration. (b) Serum IL-17F levels were not significantly elevated in pSS patients with a longer disease duration.

The relationship between pSS clinical and laboratory features and serum IL-17F and IL-17A levels were presented in Table 2, and IL-17F were positively correlated with IgG (*r* = 0.026, *P* = 0.018) and IgM (*r* = 0.215, *P* = 0.025) and negatively correlated with C3 (*r* = −0.284, *P* = 0.003) and C4 (*r* = −0.235, *P* = 0.014) (Figure 2). However, serum level of IL-17A was only negatively correlated with C3 (*r* = −0.304, *P* = 0.001) (Figure 3). Besides, patients with elevated IgG decreased C4 and moderate to high disease activity showed significantly elevated serum level of IL-17F. However, elevated serum IL-17A was only observed in patients with increased RF (data shown in Table 4). As shown in Table 3, patients with higher serum levels of IL-17F showed neutropenia, granulocytopenia, elevated IgG, and decreased C3. Increased percentage of patients with moderate to high disease activity (ESSDAI ≥ 5) was found in the group with elevated serum IL-17F. But pSS patients with higher serum levels of IL-17A showed elevated IgM and decreased C3. Interestingly, as shown in Figure 4, pSS patients with longer disease duration (≥8 years) had significantly elevated levels of IL-17A (Figure 4(a)  11.25 ± 4.70 versus 9.29 ± 2.42, *P* = 0.049). However, there were no differences in IL-17F (Figure 4(b)  21.71 ± 21.79 versus 18.69 ± 19.27, *P* = 0.163) between these two groups.

We also analyzed the correlation between the serum levels of IL-17F and IL-17A and lymph node enlargement, splenomegaly, joint involvement, lung involvement, RBC, Hb, PLT, IgA, ESR, and anti-SSB antibody and did not find any correlation between them.

These results revealed that it was IL-17F that was more correlated with increased autoantibody level and increased autoimmune conditions such as reduction of C3/4, presence of neutropenia, granulocytopenia, and elevated ESSDAI, instead of IL-17A.

## 4. Discussion

In this study, we determined and compared the serum levels of IL-17A and IL-17F and found that IL-17F, instead of IL-17A, was significantly increased in serum from pSS patients than in healthy controls. We further revealed their clinical relevance in pSS patients which suggested that different IL-17 family members might play different pathogenic roles in pSS.

Previous studies demonstrated elevated levels of IL-17A in body fluids from pSS patients such as saliva [3–11], tears [4–6, 8], and serum [4, 7, 9], which supported the pathogenic effects of IL-17 axis in pSS. It was reported that IL-17A positive pSS patients had significantly longer disease durations and less prevalent parotid gland swelling compared to those IL-17A negative ones [9]. However, despite its widely accepted pathogenic functions, no correlation between IL-17A and clinical severity or specific extraglandular manifestations has been reported [27]. In this study, we only observed slightly higher level of serum IL-17A in pSS patients compared to healthy controls without statistical significance. IL-17A was only associated with elevated RF and decreased C3 and was significantly higher in patients with longer disease duration of more than 8 years, which was consistent with previous findings [27, 28]. All these studies confirmed that the clinical relevance of IL-17A in pSS patients is not remarkable despite its notable pathogenic effects in both animal model and in vitro cell culture systems [2].

Surprisingly, we found that IL-17F, the closest homologue of IL-17A, was significantly elevated in pSS patients and correlated with hyperactivity of humeral autoimmune response, including increased higher IgG, higher IgM, higher levels of RF and higher titers of ANA, and anti-SSA antibody. Furthermore, higher levels of IL-17F were found in patient groups with neutropenia, granulocytopenia, and increased ESSDAI, which indicated that IL-17F was associated with pSS disease activity and severity. Former studies mainly focused on the pathogenic roles of IL-17A in pSS, and after failing to reveal the association between IL-17A and extraglandular involvement, they concluded that IL-17/Th17 might not be involved in clinical manifestations other than glandular impairment [2, 27]. However, involvement of the other IL-17 family cytokines such as IL-17F in autoimmune diseases is still elusive. In this study, for the first time, we revealed that IL-17F was associated with elevated autoantibody level and increased disease activity in pSS.

IL-17F is expressed by Th17 cells and other types of IL-17-expressing T cells such as *γδ*T and NKT in vivo. Many previous studies have described the similar regulation of IL-17A and IL-17F induced by IL-23, TGF-*β*, IL-6, and IL-21, as well as transcription factors ROR*γ*t and STAT3 [23]. Although there are remarkable sequence homology and functional similarity between IL-17A and IL-17F, the mechanism of their difference on biological functions is not fully understood [29]. Wright et al. found that binding affinity of IL-17F to IL-17RA is much lower than that of IL-17A [30] and only IL-17F binds to IL-17RC [31], which suggested that the receptor complexes were differentially engaged by IL-17A and IL-17F. IL-17A is shown to activate all three subgroups of mitogen-activated protein kinases, the extracellular signal-regulated kinases (ERK1 and ERK2), Jun NH2-terminal kinases, and p38 in several cell types, but IL-17F only induces the activation of ERK1/2. Due to its lower affinity, IL-17F seems to be less proinflammatory and IL-17A is more critical than IL-17F in mediating inflammation and autoimmunity, such as in experimental autoimmune encephalomyelitis (EAE) [32]. However, Yang et al. showed IL-17F was more critical than IL-17A in inducing airway inflammation and colitis [23], and Watanabe et al. found IL-17F is a stronger inducer of IL-8 released by keratinocytes and cystic fibrosis, which suggests that IL-17F might play a more prominent role in psoriasis [21, 22]. Moreover, IL-17F is associated with relapse in multiple sclerosis, and IL-17A does not show that correlation, which is quite different from animal models [33]. Similarly, our study found that IL-17F, instead of IL-17A, was significantly correlated with production of autoantibodies and disease activity in pSS. It has been revealed that in several types of tissues, including liver, lung, and ovary, only IL-17F was expressed [14, 34], so the wider tissue distribution of IL-17F might contribute to its involvement in extraglandular pathogenesis in pSS. Recently, Giles et al. discovered that besides IL-17A, IL-17F also played an important role in the driven inflammation of nonalcoholic fatty liver disease [35]. Moreover, IL-17F competed for the receptor with IL-17A and inhibited IL-17A engagement but IL-17F was not inhibited by IL-17A [34]. All these findings support that IL-17F plays a more important pathogenic role in autoimmune disease.

For the first time, we simultaneously examined the serum levels of IL-17A and IL-17F and evaluated and compared their clinical relevance in pSS. Our study demonstrated that serum levels of IL-17F significantly increased in pSS patients and were associated with elevated autoantibody level, more severe autoimmune conditions, and higher disease activity, but IL-17A was only associated with longer disease duration. Our findings suggest that different IL-17 family members might play different roles in the onset and exacerbation of pSS. Further investigations on the exact mechanisms of such discrepancy will provide novel insights into the roles of IL-17 family cytokines in pSS pathogenesis and reveal potential therapeutic value of IL-17 family members.

## Figures and Tables

**Table 1:** Characteristics of the studied 109 pSS patients and 42 healthy controls.

Clinical characteristics	pSS	Controls	*t*/*χ* ^2^	*P*
Age	55.72 ± 14.64	55.12 ± 13.17	0.231	0.818
Sex (F : M)	105 : 4	40 : 2	0.095	0.670
WBC (×10^9^/L)	4.77 ± 2.10	—	—	—
Neutrophils (×10^9^/L)	3.04 ± 1.89	—	—	—
RBC (×10^12^/L)	3.74 ± 0.62	—	—	—
Hb (g/L)	116.17 ± 18.19	—	—	—
PLT (×10^9^/L)	143.28 ± 70.95	—	—	—
IgA (g/L)	3.37 ± 1.77	—	—	—
IgG (g/L)	19.44 ± 9.45	—	—	—
IgM (g/L)	1.68 ± 1.33	—	—	—
C3 (g/L)^*∗*^	0.90 ± 0.21	—	—	—
C4 (g/L)^*∗*^	0.19 ± 0.07	—	—	—
ESR (mm/h)	32.82 ± 30.12	—	—	—
RF (IU/mL)^*∗*^	178.65 ± 295.04	—	—	—
ANA ≥ 1 : 320^*∗∗*^	56.31%	—	—	—
Anti-SSA ≥ 200 RU/mL	62.85%	—	—	—
Anti-SSB ≥ 20 RU/mL	55.06%	—	—	—
ESSDAI ≥ 5	56.88%	—	—	—

^*∗*^1 patient did not have the data of C3 and C4.

^*∗∗*^4 patients did not have the data of ANA.

**Table 2:** Correlation of serum IL-17F and IL-17A with clinical and laboratory features of pSS patients.

Clinical manifestations and laboratory features	IL-17F		IL-17A	
	Spearman *r*	*P*	Spearman *r*	*P*
WBC	−0.146	0.129	−0.135	0.162
Neutrophils	−0.116	0.23	−0.086	0.376
RBC	0.056	0.561	−0.029	0.765
Hb	0.01	0.918	−0.067	0.491
PLT	0.062	0.521	0.039	0.686
IgA	0.015	0.88	−0.055	0.572
IgG	**0.226**	**0.018**	−0.039	0.684
IgM	**0.215**	**0.025**	0.107	0.27
C3	**−0.284**	**0.003**	**−0.304**	**0.001**
C4	**−0.235**	**0.014**	−0.179	0.064
ESR	−0.054	0.574	0.035	0.72
RF	**0.481**	**<0.001**	**0.234**	**0.015**
Anti-SSA	**0.236**	**0.014**	−0.056	0.565
Anti-SSB	0.039	0.691	−0.148	0.125

**Table 3:** Clinical and laboratory characteristics of pSS patients with the elevated and normal levels of serum IL-17F and IL-17A.

Clinical and laboratory parameters	IL-17F				IL-17A			
	<15.44 pg/mL (*n* = 66)	≥15.44 pg/mL (*n* = 43)	*t*/*u*/*χ* ^2^	*P*	<13.8 pg/mL (*n* = 97)	≥13.8 pg/mL (*n* = 12)	*t*/*u*/*χ* ^2^	*P*
Lymphadenectasis	15 (22.73%)	10 (23.26%)	0.004	0.949	20 (20.62%)	5 (41.67%)	2.677	0.102
Splenomegaly	8 (12.12%)	5 (11.63%)	0.006	0.938	11 (11.34%)	2 (16.67%)	—	0.634
Joint involvement	6 (9.09%)	6 (13.95%)	0.628	0.535	12 (12.37%)	0	—	0.354
Lung involvement	32 (48.48%)	20 (46.51%)	0.041	0.84	48 (49.48%)	4 (33.33%)	—	0.366
WBC (×10^9^/L)	4.39 (3.59, 6.44)	3.72 (3.02, 5.00)	**1.975**	**0.048**	4.20 (3.14, 5.95)	4.26 (3.71, 5.66)	0.239	0.812
Neutrophils (×10^9^/L)	2.55 (1.79, 4.45)	2.18 (1.61, 3.41)	**2.309**	**0.023**	2.39 (1.69, 3.82)	2.31 (1.71, 3.97)	0.254	0.8
RBC (×10^12^/L)	3.71 ± 0.65	3.77 ± 0.57	−0.543	0.588	3.74 ± 0.62	3.71 ± 0.69	0.167	0.867
Hb (g/L)	117.17 ± 17.28	114.63 ± 19.61	0.71	0.479	116.94 ± 16.81	109.93 ± 27.09	1.263	0.209
PLT (×10^9^/L)	142.59 ± 78.08	144.33 ± 59.23	−0.124	0.901	142.79 ± 71.57	147.17 ± 65.58	−0.201	0.841
IgA (g/L)	2.78 (2.07, 4.21)	3.25 (2.27, 4.25)	−0.682	0.495	3.37 ± 1.73	3.45 ± 2.17	−0.044	0.965
IgG (g/L)	17.48 ± 9.19	22.46 ± 9.15	−**2.2772**	**0.007**	19.30 ± 9.56	20.58 ± 8.85	−0.842	0.4
IgM (g/L)	1.13 (0.78, 2.03)	1.43 (1.07, 2.18)	−1.779	0.075	1.62 ± 1.30	2.21 ± 1.52	−**1.965**	**0.049**
C3 (g/L)	0.94 ± 0.23	0.85 ± 0.17	**2.228**	**0.028**	0.92 ± 0.22	0.81 ± 0.13	**2.093**	**0.036**
C4 (g/L)	0.19 ± 0.07	0.18 ± 0.06	1.355	0.178	0.19 ± 0.06	0.16 ± 0.08	1.336	0.181
ESR (mm/h)	32.33 ± 29.44	33.56 ± 31.47	−0.207	0.837	32.06 ± 29.68	38.92 ± 34.28	−0.742	0.46
RF (IU/mL)	20 (20, 69.50)	205.00 (42.00, 462.00)	−**3.594**	**0.001**	27.35 (20.00, 193.50)	345.00 (24.28, 573.25)	−**2.419**	**0.016**
ANA ≥ 1 : 320	30 (46.15%)	28 (70%)	**5.695**	**0.026**	30 (31.91%)	8 (72.73%)	**6.948**	**0.017**
Anti-SSA (RU/mL)	454.25 (18.89, 1386.96)	1484.99 (178.52, 1689.50)	−**3.225**	**0.002**	914.45 (66.15, 1618.97)	176.97 (3.91, 1567.35)	1.081	0.282
Anti-SSB (RU/mL)	15.51 (2.16, 199.63)	15.74 (4.05, 250.09)	−0.967	0.337	17.19 (2.33, 228.81)	7.19 (4.27, 191.45)	0.513	0.609
ESSDAI ≥ 5	33 (50%)	30 (69.77%)	**4.171**	**0.041**	55 (56.70%)	8 (66.67%)	0.435	0.51

**Table 4:** Serum levels of IL-17A and IL-17F in the presence and absence of clinical and laboratory manifestations of pSS patients.

Clinical and laboratory parameters	IL-17F (pg/mL)				IL-17A (pg/mL)			
	Presence	Absence	*t*/*u*/*χ* ^2^	*P*	Presence	Absence	*t*/*u*/*χ* ^2^	*P*
Lymphadenectasis	12.68 (8.69, 20.08) (*n* = 25)	13.09 (10.00, 21.54) (*n* = 84)	−0.519	0.604	9.13 (7.88, 13.38) (*n* = 25)	9.75 (8.51, 10.36) (*n* = 84)	−0.268	0.789
Splenomegaly	12.68 (9.22, 22.69) (*n* = 13)	13.09 (9.60, 20.26) (*n* = 96)	0.164	0.87	9.75 (9.13, 11.59) (*n* = 13)	9.44 (7.88, 10.36) (*n* = 96)	1.618	0.106
Joint involvement	14.89 (9.87, 22.81) (*n* = 12)	12.68 (9.73, 19.98) (*n* = 97)	0.581	0.561	7.88 (7.88, 9.13) (*n* = 12)	9.75 (8.51, 10.98) (*n* = 97)	−2.443	0.015
Lung involvement	12.81 (10.00, 19.42) (*n* = 52)	13.22 (9.47, 27.27) (*n* = 57)	−0.525	0.6	9.44 (8.51, 10.59) (*n* = 52)	9.75 (7.88, 10.98) (*n* = 57)	−0.091	0.927
WBC ≤ 3.5 × 10^9^/L	17.13 (9.60, 34.15) (*n* = 32)	12.68 (9.73, 19.42) (*n* = 77)	1.535	0.125	9.75 (9.13, 11.36) (*n* = 32)	9.13 (7.88, 10.67) (*n* = 77)	1.128	0.259
Neutrophils ≤ 1.8 × 10^9^/L	15.16 (8.69, 27.27) (*n* = 33)	12.68 (10.00, 19.42) (*n* = 76)	0.521	0.602	9.75 (8.51, 10.97) (*n* = 33)	9.44 (7.88, 10.90) (*n* = 76)	0.386	0.998
RBC ≤ 3.5 × 10^12^/L	13.23 (9.47, 28.07) (*n* = 57)	12.95 (10.00, 18.85) (*n* = 52)	0.747	0.455	9.44 (7.88, 10.51) (*n* = 57)	9.75 (7.88, 11.21) (*n* = 52)	−0.679	0.497
Hb ≤ 110 g/L	12.14 (8.95, 17.99) (*n* = 35)	13.77 (9.87, 28.88) (*n* = 74)	−1.315	0.189	9.75 (7.88, 10.98) (*n* = 35)	9.59 (7.88, 10.51) (*n* = 74)	0.199	0.843
PLT ≤ 100 × 10^9^/L	12.95 (9.47, 22.07) (*n* = 53)	13.22 (10.00, 20.26) (*n* = 56)	−0.363	0.718	9.13 (7.88, 10.51) (*n* = 53)	9.75 (8.11, 10.98) (*n* = 56)	−0.183	0.855
IgA ≥ 4.5 g/L	13.77 (10.00, 29.61) (*n* = 23)	12.68 (9.47, 19.70) (*n* = 86)	0.353	0.725	9.75 (7.88, 11.59) (*n* = 23)	9.59 (7.88, 10.36) (*n* = 86)	0.927	0.362
IgG ≥ 16 g/L	15.44 (10.00, 27.44) (*n* = 60)	12.14 (9.21, 17.14) (*n* = 49)	**2.06**	**0.039**	9.13 (7.88, 10.98) (*n* = 60)	9.75 (8.19, 10.36) (*n* = 49)	−0.59	0.555
IgM ≥ 3 g/L	14.05 (10.13, 27.77) (*n* = 12)	12.68 (9.47, 19.42) (*n* = 97)	0.722	0.471	10.67 (9.13, 11.59) (*n* = 12)	9.44 (7.88, 10.36) (*n* = 97)	1.928	0.054
C3 < 0.78 g/L	14.33 (11.60, 22.69) (*n* = 29)	12.14 (9.47, 19.42) (*n* = 80)	1.158	0.247	9.75 (8.82, 11.59) (*n* = 29)	9.13 (7.88, 10.67) (*n* = 80)	1.586	0.113
C4 < 0.17 g/L	14.88 (12.14, 29.61) (*n* = 39)	11.60 (8.95, 18.99) (*n* = 70)	**2.525**	**0.012**	9.75 (9.13, 11.59) (*n* = 39)	9.44 (7.88, 10.36) (*n* = 70)	1.816	0.069
ESR ≥ 20 mm/h	13.49 (10.00, 22.90) (*n* = 56)	12.68 (9.47, 19.98) (*n* = 53)	0.522	0.603	9.75 (7.88, 11.51) (*n* = 56)	9.44 (7.88, 10.36) (*n* = 53)	0.996	0.321
RF ≥ 20 IU/mL	18.27 (11.06, 32.07) (*n* = 61)	10.53 (8.44, 13.77) (*n* = 48)	**4.407**	**<0.001**	9.75 (8.19, 11.59) (*n* = 61)	9.13 (7.88, 10.06) (*n* = 48)	**2.637**	**0.01**
Anti-SSA ≥ 200 RU/mL	14.88 (10.00, 25.03) (*n* = 68)	10.53 (8.69, 16.71) (*n* = 41)	**2.375**	**0.018**	9.59 (8.04, 10.36) (*n* = 68)	9.75 (7.88, 11.59) (*n* = 41)	−0.03	0.976
Anti-SSB ≥ 20 RU/mL	21.00 ± 19.95 (*n* = 50)	19.80 ± 21.39 (*n* = 59)	0.304	0.762	9.95 ± 4.16 (*n* = 50)	10.73 ± 3.77 (*n* = 59)	−1.019	0.311
ANA ≥ 1 : 320	14.88 (10.00, 29.61) (*n* = 58)	11.06 (9.47, 18.27) (*n* = 47)	**2.132**	**0.033**	9.44 (8.35, 11.13) (*n* = 58)	9.44 (7.88, 10.36) (*n* = 47)	0.29	0.772
ESSDAI ≥ 5	14.60 (10.53, 22.27) (*n* = 62)	10.53 (8.95, 19.42) (*n* = 47)	**2.109**	**0.035**	9.44 (7.88, 10.98) (*n* = 62)	9.75 (7.88, 10.36) (*n* = 47)	0.021	0.983
